# Reference values for the 6-minute walk test in healthy children and adolescents in Switzerland

**DOI:** 10.1186/1471-2466-13-49

**Published:** 2013-08-05

**Authors:** Silvia Ulrich, Florian F Hildenbrand, Ursula Treder, Manuel Fischler, Stephan Keusch, Rudolf Speich, Margrit Fasnacht

**Affiliations:** 1 Department of Heart, Vessel, Thorax, University Hospital of Zurich, Switzerland and Centre of Integrative Human Physiology, Zurich, Switzerland; 2 Research Fellow Pulmonary Hypertension Unit, Department of Heart, Vessel, Thorax, University Hospital of Zurich, Zurich, Switzerland; 3 Department of Internal Medicine and Oncology, Pulmonary hypertension unit, University Hospital of Zurich, Zurich, Switzerland; 4 Department of Internal Medicine, City Hospital Waid, Zurich, Switzerland; 5 Research Fellow Pulmonary Hypertension, Unit Department of Heart, Vessel, Thorax, University Hospital of Zurich, Zurich, Switzerland; 6 Department of Internal Medicine and Oncology, University Hospital of Zurich, Zurich, Switzerland; 7 Paediatric Clinic, University Children’s Hospital Zurich, Zurich, Switzerland; 8 Respiratory Clinic, University Hospital of Zurich, Rämistrasse 100, 8091 Zürich, Switzerland

**Keywords:** 6-minute walk test, Healthy children, Exercise physiology, Exercise testing, Predicted equation, Reference value

## Abstract

**Background:**

The six-minute walk test (6MWT) is a simple, low tech, safe and well established, self-paced assessment tool to quantify functional exercise capacity in adults. The definition of normal 6MWT in children is especially demanding since not only parameters like height, weight and ethnical background influence the measurement, but may be as crucial as age and the developmental stage. The aim of this study is establishing reference values for the 6MWT in healthy children and adolescents in Switzerland and to investigate the influence of age, anthropometrics, heart rate, blood pressure and physical activity on the distance walked.

**Methods:**

Children and adolescents between 5–17 years performed a 6MWT. Short questionnaire assessments about their health state and physical activities. anthropometrics and vitals were measured before and after a 6-minute walk test and were previously defined as secondary outcomes.

**Results:**

Age, height, weight and the heart rate after the 6MWT all predicted the distance walked according to different regression models: age was the best single predictor and mostly influenced walk distance in younger age, anthropometrics were more important in adolescents and females. Heart rate after the 6MWT was an important distance predictor in addition to age and outreached anthropometrics in the majority of subgroups assessed.

**Conclusions:**

The 6MWT in children and adolescents is feasible and practical. The 6MWT distance depends mainly on age; however, heart rate after the 6MWT, height and weight significantly add information and should be taken into account mainly in adolescents. Reference equations allow predicting 6-minute walk test distance and may help to better assess and compare outcomes in young patients with cardiovascular and respiratory diseases and are highly warranted for different populations.

## Background

The six-minute walk test (6MWT) is a simple, low tech, safe and well established self-paced assessment tool to quantify functional exercise capacity in individual with various cardiorespiratory diseases [1]. The 6MWT is performed by instructing the participant to walk as fast as possible (without running) on a flat surface in 6 minutes, the distance walked (6MWD) is recorded [1]. The 6MWD is a good predictor for morbidity and mortality in various diseases in adult populations [2-6]. Normal values available for the 6MWD are based on different adult cohorts [7-9]. From these, patients’ 6MWD in % predicted of normal values can be calculated by formulas. However, whether the expression of 6MWD in % predicted provide better prognostic information is still debated [9].

The definition of normal 6MWD in children is especially demanding since not only parameters like height, weight and ethnical background influence the measurement, but may be as crucial as age and the developmental stage [10]. Therefore a reliable comparison with healthy children of similar age, ethnical background and posture is important. Values for children living in different countries may not directly applicable to each other [5]. Until now, data on normal values of the 6MWD performed according to ATS standards for healthy children and adolescence of both sex living in Central Europe are very rare and highly warranted in order to have comparative data for children and adolescents with disease. Previous studies assessing normal values for the 6MWD in children or adolescent mostly investigated restricted age ranges, distinct ethnics, were limited by their small number of participants or investigated modified 6MWD tests [10-12].

The aim of the present study was to provide reference values of the 6MWD based on demographics for children and adolescents, aged 5–17 years living in Switzerland. Further aims were to study the differential influence of age, sex, height and weight on the 6MWD and the potential added values of arterial blood pressure (BP), heart rate (HR) before and after the 6MWD and questionnaire-assessed physical activity in everyday life. We aimed to study the above-mentioned measures taken as a whole, gender-specific and separately for children and adolescents as we hypothesised that age, sex and anthropometrics would influence the distance walked.

## Methods

### Patients

This study was performed according to the Declaration of Helsinki, ICH-GCP as well as all national legal and regulatory requirements. The study was approved by the local ethics committee (Ethics committee University of Zurich). The authors of this manuscript comply with the Principles of Ethical Publishing in the International Journal of Cardiology [13]. Written informed consent for all participants was given by parents or legal guardian as well as by subjects. Recruitment took place at Swiss schools in rural and urban areas after contacting the authorities by an information letter and through mouth-to-mouth propaganda. All candidates asked to participate took part in this study. Questionnaire assessment and 6MWT were performed by the same two study nurses in all participants.

### 6 minute walk test

Questionnaire and 6MWT took place at the same day. In brief, exact age and sex were noted. Body weight and height were measured and the body mass index (BMI, in kg/m^2^) was calculated by dividing the weight (in kg) by the square of the body height in meters (m). Thereafter, the blood pressure (mmHg), heart rate (bpm) and the peripheral arterial oxygen saturation (SpO_2_%) were measured after a resting period in sitting position for at least 5 minutes. Thereafter the 6MWT was performed according to the guidelines of the American Thoracic Society for adults [1]. Children and adolescents were instructed according to a standardised protocol to perform the 6MWT by walking around two flagpoles positioned 30 m apart on a flat ground. Subjects were instructed to walk as fast as possible (without running) at a steady pace for 6 minutes. After 5 minutes time left had been advised to the participant. No other commandos or verbal feedback was given. Specially trained study nurses who supervised the test measured the exact covered distance. Directly after the test, exercise BP, HR and SpO_2_ were re-measured. The mean arterial pressure (MAP) was calculated by adding twice the diastolic pressure to the systolic pressure and dividing the sum by three.

### Questionnaire assessments

Health state of participants was assessed by a standardised questionnaire asking the following information from parents and adolescents, respectively: Is your child/are you healthy? Is your child/are you able to participate normally in physical activities? Is your child/are you taking regular medication? Does your child/do you suffer from asthma or another chronic disease? Did your child/did you have an operation of the lower extremities, which make it difficult for you to walk? If with the aid of this questionnaire disability was detected, the child was excluded from the study. In addition, we asked for physical activities as follows: How many hours do your child/do you participate in physical activities a week beside the obligatory school sport? Answers were scored referred to physical activity score (PAS) on a scale between 0 and 4 with 0 standing for no additional physical activity, 1 for 1–2, 2 for 3–4 and 4 for > 4 hours of weekly physical activity.

### Statistical methods and power calculation

Power calculation on the basis of the available data from adults [7] revealed that with the assumption of a standard deviation of 40 m per age group the inclusion of 20 girls and boys each for each age year resulting in testing a total of 440 children respectively adolescents would be sufficient to provide reliable normal values of the 6MWD for the age groups tested including an alpha error of 5%. Characteristics of the participants are summarized by means ± (standard deviation, SD). Patients were allocated into yearly age groups and binary by an assumed onset of puberty at 12 years in girls and 13 years in boys in children and adolescents. Baseline variables for different groups have been compared using Mann–Whitney-*U*-Test. Percentile curves were plotted using a specialized computer program (LMSchartmaker pro, Version 2.43, http://homepage.mac.com/tjcole). Pearson correlation, linear and step-wise multiple linear regressions as provided by the SPSS program were used to correlate characteristics with the 6MWD and to provide prediction equations for the 6MWD. Durbin-Watson tests (DW) were used to correct for autocorrelation in multiple linear regression models. IBM, SPSS package Version 21 was used for statistical calculation. A probability of P < 0.05 has to be considered significant.

## Results

### General

A total of 496 children and adolescents (252 girls) were included upon written informed consent. All participants were healthy according to the predefined questionnaire. Subjects’ characteristics are shown in Table 1. All children completed the entire 6MWT according to the protocol and thus none of them had to be excluded from the study. In no case it was necessary to stop the test prematurely, and there were no unexpected events during the tests.

**Table 1:** Patients’ characteristics I

	**Numbers (%)**
	**Mean ± SD**
**Total number of patients**	496
**Female/male**	252/244 (51/49)
**Age (years)**	11.1 ±3.4
**Body weight (kg)**	41 ±17
**Height (cm)**	147 ±2
**Body mass index (kg/m**^**2**^**)**	17.9 ±3.3
Mean Artery Pressure before test (mmHg)	80 ±10
Mean Artery Pressure after test (mmHg)	87 ±11
Pulse rate before test (bpm)	95 ±16
Pulse rate after test (bpm)	136 ±23
Pulse rate increase (bpm)	41 ±23
Transcutaneous oxygen saturation before test (%)	97 ±4
Transcutaneous oxygen saturation after test (%)	97 ± 1
Desaturation (%)	0 ± 1
Six-minute walking distance (m)	618 ± 79

### Gender-related characteristics in 6 minute walk distance, body dimensions and vital signs

**Figure 1:**
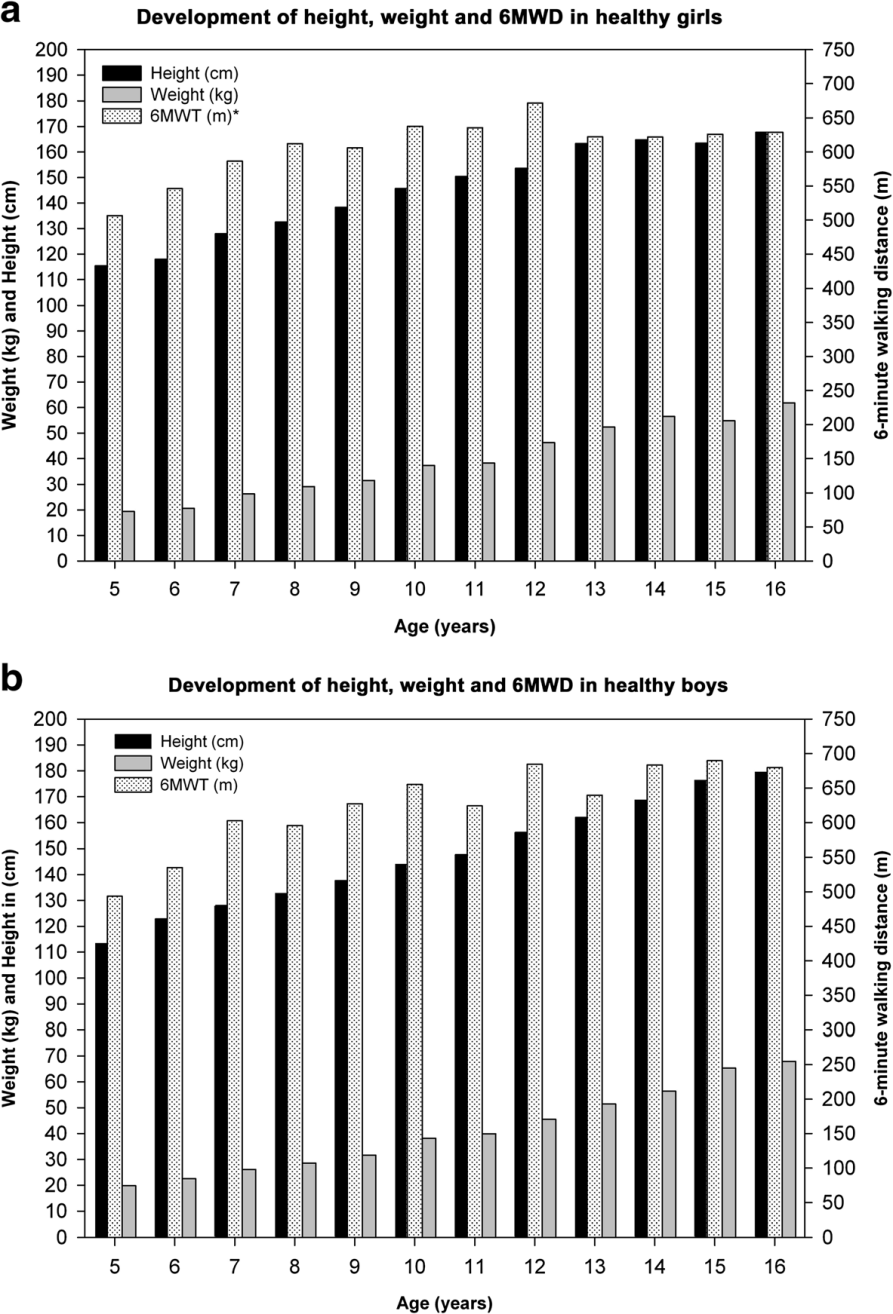
**Shows the development of gender-related anthropometrics in healthy children between 5 to 16 years of age. (a)**. Association between 6-minute walking distance (6MWD), height, weight and age in healthy Caucasian girls and **(b)**. Association between 6-minute walking distance (6MWD), height, weight and age in healthy Caucasian boys.

**Figure 2:**
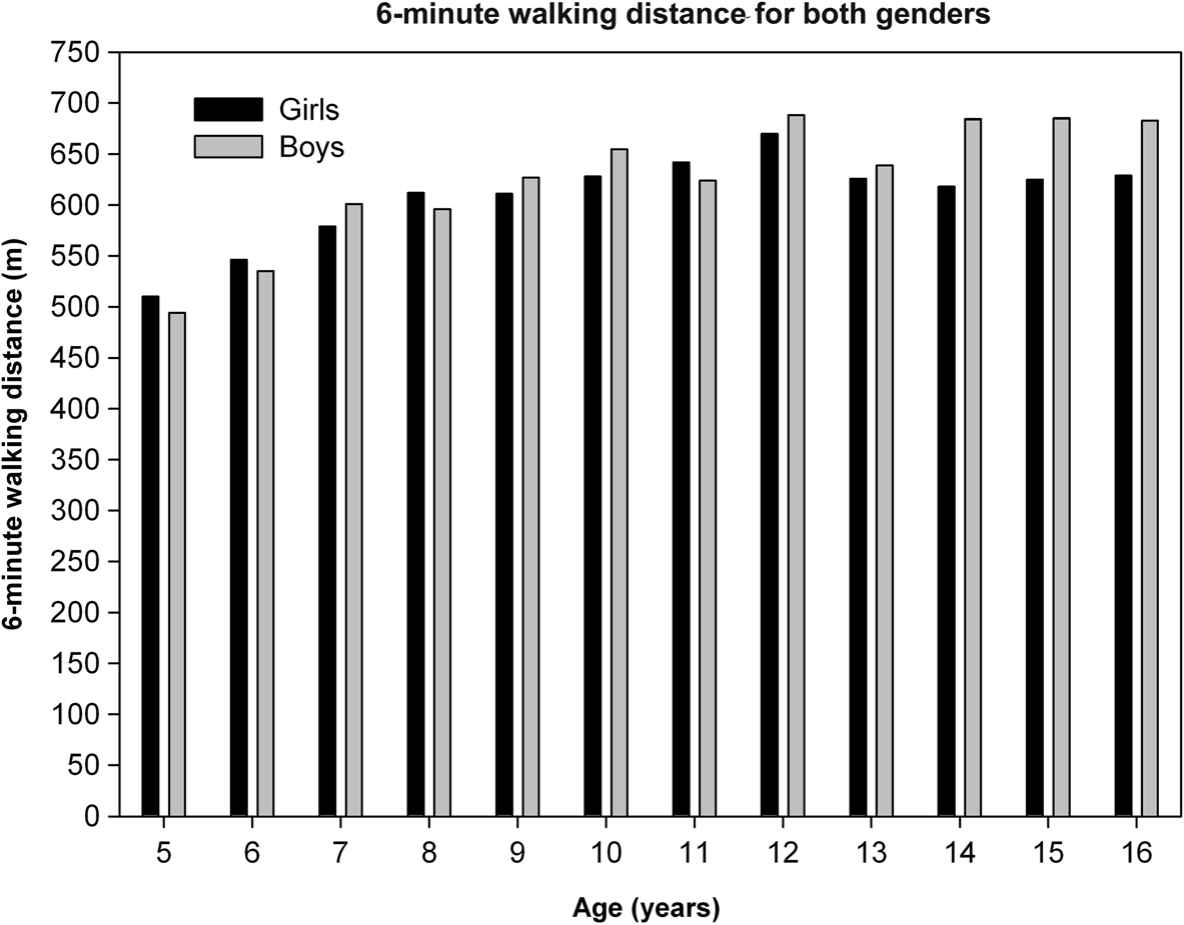
**Association between 6-minute walking distance (6MWD) and age in healthy Caucasian girls and boys.**

Overall, the mean distance walked within six minutes was 618 ± 79 meters. Whereas weight and height steadily increased with age, the 6MWD mainly increased until puberty and then flattened (Figure 1a and b). 6MWD distances, vital signs by gender-specific yearly age groups are shown in Table 2 and Figure 2.

**Table 2:** Age stratified characteristics and Six-minute walk test

							**Before 6MWT**		**After 6MWT**	
**Sex**	**Age categroy (year)**	**n (%)**	**Height (cm)**	**Weight (kg)**	**BMI (kg/m**^**2**^)*	6MWT (m)*	MAP (mmHg)*	HR (bpm)*	MAP* (mmHg)	HR* (bpm)
**Male**	5	19 (8)	113 ±6	20 ±3	15.5 ±2.3	494 ± 60	70 ±7	104 ±19	77 ±8	132 ±20
	6	22 (9)	123 ±5	23 ±3	14.8 ±1.6	535 ± 73	72 ±8	100 ±15	80 ±10	122 ±30
	7	19 (8)	128 ±6	26 ±4	15.8 ±1.7	603 ± 51	74 ±5	96 ±15	83 ±6	131 ±21
	8	22 (9)	133 ±7	29 ±4	16.1 ±1.1	596 ± 59	79 ±8	96 ±17	85 ±10	129 ±21
	9	18 (7)	138 ±6	32 ±4	16.6 ±1.6	627 ± 70	77 ±7	94 ±12	84 ±9	138 ±19
	10	19 (8)	144 ±7	38 ±8	18.2 ±2.5	655 ± 53	82 ±9	97 ±14	95 ±7	141 ±25
	11	23 (9)	148 ±6	40 ±10	18.1 ±3.8	624 ± 87	80 ±9	94 ±12	88 ±10	139 ±20
	12	20 (8)	156 ±9	46 ±10	18.4 ±2.6	685 ± 74	82 ±7	93 ±9	89 ±9	145 ±30
	13	21 (9)	162 ±9	51 ±10	19.5 ±2.9	639 ± 49	83 ±9	96 ±15	87 ±8	129 ±18
	14	20 (8)	169 ±6	56 ±10	19.7 ±2.8	684 ±81	86 ±9	98 ±20	94 ±6	138 ±25
	15	20 (8)	176 ±7	65 ±13	20.9 ±3.8	690 ±71	92 ±10	103 ±24	100 ±9	141 ±24
	16	21 (9)	180 ±9	68 ±13	20.9 ±3.2	680 ±55	93 ±10	88 ±16	100 ±10	135 ±28
**Female**	5	19 (8)	115 ±5	19 ±3	14.5 ±1.5	506 ±39	71 ±7	95 ±17	77 ±6	129 ±17
	6	21 (8)	118 ±6	21 ±4	14.7 ±1.6	546 ±51	69 ±6	94 ±21	79 ±8	137 ±17
	7	19 (8)	128 ±6	26 ±5	16 ±2.4	586 ±59	71 ±8	99 ± 14	82 ±7	132 ±20
	8	21 (8)	133 ±7	29 ±6	16.5 ±1.9	612 ±40	78 ±7	96 ±17	84 ±12	130 ±17
	9	18 (7)	138 ±5	32 ±4	16.4 ±1.8	606 ±52	77 ±11	96 ±19	87 ±9	139 ±18
	10	22 (9)	146 ±7	37 ±8	17.5 ±3.0	638 ±63	78 ±9	98 ±14	82 ±11	140 ±22
	11	20 (8)	150 ±8	38 ±10	16.7 ±2.6	636 ±54	80 ±7	91 ±17	83 ±8	148 ±19
	12	20 (8)	154 ±8	46 ±9	19.5 ±2.7	672 ±55	82 ±8	94 ±12	92 ±9	153 ±21
	13	27 (11)	163 ±7	52 ±10	19.6 ±3.1	622 ±76	82 ±9	96 ±14	88 ±9	135 ±25
	14	23 (9)	165 ±7	56 ±8	20.8 ±2.4	622 ±64	85 ±7	90 ±18	90 ±10	132 ±24
	15	22 (9)	163 ±6	55 ±10	20.5 ±2.9	626 ±49	89 ±12	97 ±19	95 ± 12	139 ±20
	16	20 (8)	168 ±7	62 ±9	21.8 ±2.4	629 ±52	85 ±8	88 ±14	97 ±10	134 ±17

Age stratified characteristics and 6-minute walking test in male and female participants. * Values presented as mean ± *SD. BMI* (kg/m^2^), Body mass index, *MAP* mean arterial blood pressure (mmHg); *HR* Heart rate (beats/minute); *SpO*_*2*_ Transcutanous oxygen saturation (%), 6MWT, 6-minute walk test.

In females, the increase in 6MWD peaked just at the time of assumed puberty (around age 12) and then decreased back to a level already reached with about 9 years. In boys, the peak was similarly found around age 12, however, with exception of a temporary drop with 13, they kept the peak 6MWD reached (Figure 2). Further analysis revealed that taking a cut-off point a year earlier or later would not have changed the main prediction.

We found a significant difference between boys and girls in the 6MWD (626 ± 65 m versus 608 ± 55 m) and the PAS (3 versus 2) (p < .001 and .017, respectively). We found no other overall gender-specific differences. Separate analysis for children < 12 and ≥ 12 years revealed significant difference in the 6MWD and PAS only for adolescents (p = .000 for both). Analysis by yearly age groups revealed significant gender-specific difference in the 6MWD for 14, 15 and 16 year old and PAS-difference for 9, 12, 13, 14 and 15 years old (lower 6MWD and PAS in females).

**Figure 3:**
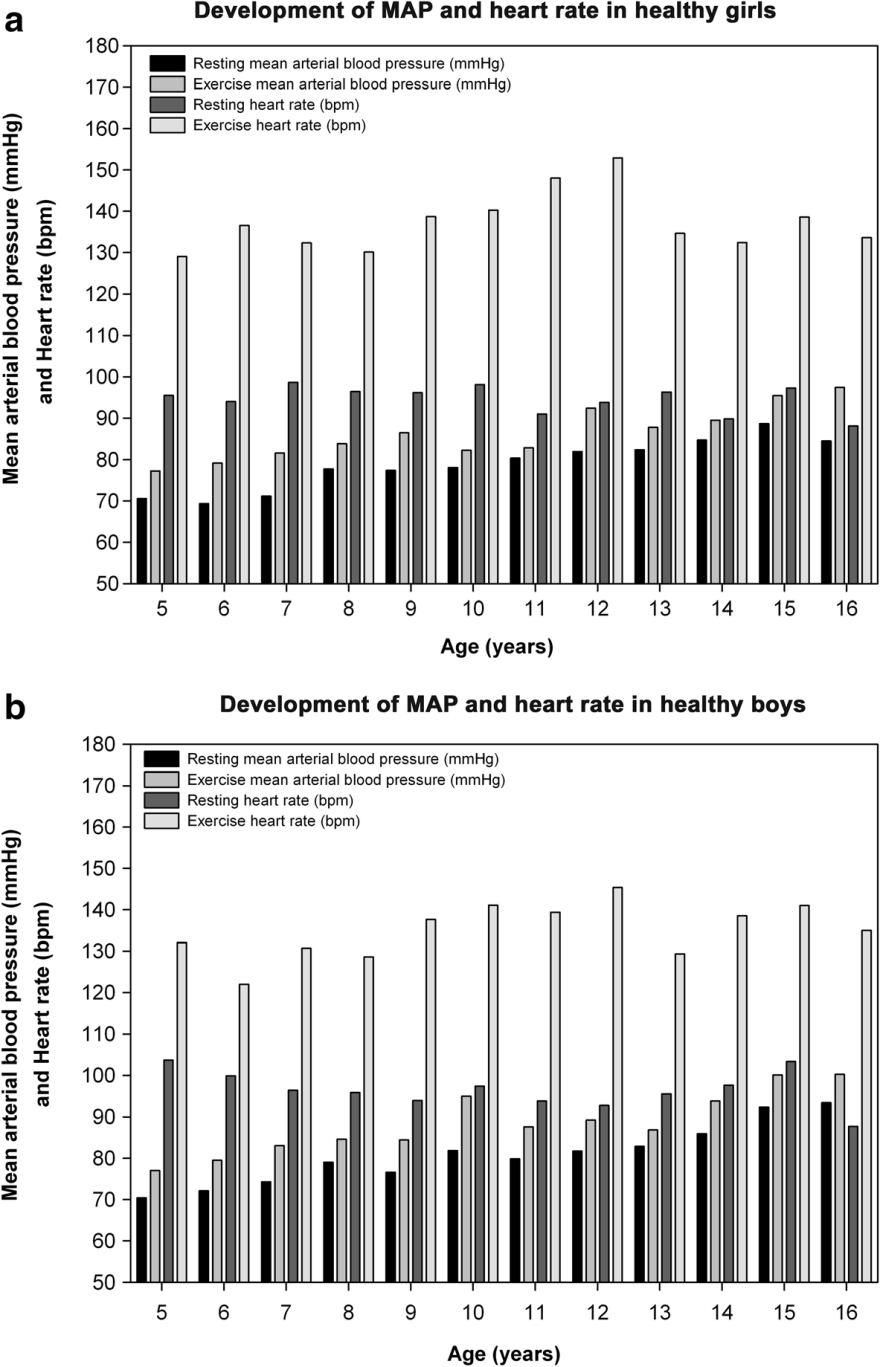
**Shows the development of gender-related hemodynamics in healthy children between 5 to 16 years of age. (a)**. Association between mean arterial blood pressure (MAP) and heart rate during rest and exercise in different age groups of healthy Caucasian girls. **(b)**. Association between mean arterial blood pressure (MAP) and heart rate during rest and exercise in different age groups of healthy Caucasian boys.

The MAP increased with age, whereas the heart rate overall slightly decreased with increasing age groups (Figure 3a and 3b).

The MAP slightly increased with exercise, on average 7 mmHg, without any significant gender or age differences. The mean heart rate at the end of the 6MWT was 136 ± 23 bpm. The HR considerably increased by a mean of 41 ± 22 bpm (43% change from resting heart rate) without major differences between age groups. We found a slightly higher exercise increase in HR in girls compared with boys (43 vs. 40 bpm, p = .025). The SpO_2_ remained stable in both sex over all ages and after exercise.

### Correlations and equations to calculate normal 6MWD in healthy children

#### Total group

We found a significant univariate correlation of age, height, weight, BMI, resting and peak exercise blood pressures, exercise HR and PAS with the 6MWD (all p < .0001). Linear regression revealed the following formula to calculate overall age-adjusted 6MWD: 6MWD = 11.89 × age (y) + 486.1 (p = .000, DW 2.045, see Table 3). Multiple, stepwise linear regression in a model including age, weight and height revealed that in this model age did no longer add information to mere weight and height. Normal values for the 6MWD could therefore be calculated from height and weight as follows (overall anthropometric formula): 6MWD = 391.9 × height (m) – 2.41 × weight (kg) + 140.2 (p = .000, DW 2.032). The same regression models were then calculated separated by gender and sub-divided by an assumed puberty of 12 years in girls and 13 years in boys (Table 3).

**Table 3:** Equations to predict the 6 minute walk distance in children and adolescents

**Model**	**Age-adjusted**	**p**	**+ height-, weight- adjusted**	**p**	**+ vitals and physical activity score (PAS)**	**p**
**Population**	**(age in years (y))**		**(age in y, height in m, weight in kg)**		**(heart rate (HR) in bpm, PAS 1-4)**	
**General**	11.89 *y + 486.1	.000, .000	391.9*m -2.41* kg + 140.2	.000, .000, .000	192.69*m + 1.27*exHR + 161.55	.000, .000, .000
**Males**	15.36 *y + 456.92	.000, .000	13.40*y -2.16*kg + 196.53*m + 276.92	.001, .001, .017, .000	14.38*y + 1.21*exHR – 2.12*kg + 166.66*m + 146.56	.000, .000, .001, .037
**<13 y**	24.18*y + 385.18	.000, .000	As age-adjusted		28.62*y + 1.26*exHR -2.034*kg + 239.29	.000, .000, .014, .000,
≥**13 y**	13.08*y + 476.69	.031, .000	As age-adjusted		1.01*exHR + 13.3*y + 338.25	.001, .022, .000
**Females**	8.623*y + 513.7	.000, .000	372.3*m -2.635*kg + 172.05	.000, .000, .001	152.58*m + 1.38*exHR + 197.97	.000, .000, .000
**<12 y**	20.83*y + 413.94	.000, .000	330.07*m + 153.3	.000, .000	279.5*m + .87*exHR + 102.45	.000, .000, .024
≥ **12 y**	-8.66*y + 757.42	.000, .036	-1.867 * kg + 734.29	.001, .000	1.79*exHR – 1.28*restHR – 2.55*kg + 203.3*m + 7.83*PAS + 298.6	.000, .000, .000, .023, .032, .030

*multiplied by, *Y* years, *m* meters of body height, *kg* kilogram body weight, *HR* heart rate, *restHR* heart rate before the 6MWD, *exHR* HR after the 6MWD. *PAS* physical activity score as measure of self-reported activity going from 0 (0 hours weekly of physical activity beside school sports to >4 hours of physical activity) to 4 P values are given for all parameters added in the equation model in order that the reader can see the impact of each measure to the equation.

### Equations by gender

In girls, we found a significant univariate correlation of age, height, weight, BMI, resting and exercise blood pressure and HR with the 6MWD (all p = .000), but not with PAS. Linear regression revealed significant age dependency of the 6MWD for girls (p = .000, DW 2.187, Table 3). Addition of weight and height revealed that age did no longer add information. Thus, a 7 year old girl being 1.25 m tall and weighing 25 kg would have a predicted walk distance of 575 m by the gender-specific age-adjusted equation, 572 m by the gender-specific anthropometric equation, and 569 m by both, the age-adjusted and anthropometric overall equation.

In boys, we found a significant univariate correlation of age, height, weight, BMI, baseline and exercise BP, exercise HR and the PAS with the 6MWD (all p < .000). The predicted 6MWD in boys could be calculated by age (p = .000, DW 1.709, Table 3). Addition of weight and height revealed that both significantly added to the equation (p = .001, .001 and .017, respectively, DW 1.724, Table 3).

### Differential equations before versus after puberty in girls and boys

In girls <12 years, linear regression revealed that age significantly predicted the 6MWD (p = .000, DW 1.901, Table 3). Multiple stepwise linear regressions by adding weight and height reveal that only height did significantly count below the age of 12 years in girls (p = .000, DW 1.928). Thus, the predicted 6MWD for a 7-year-old girl being 1.25 m tall would herewith be 566 m.

In female adolescents’ ≥12 years, age was still a significant predictor of the 6MWD, however, the significance is lower (p = .036, DW 1.901). Addition of height and weight revealed that only weight remained significant (p = .001, DW 1.870).

A 14-year-old girl being 1.65 m tall and weighting 56 kg would have a predicted walk distance of 630 m (to compare: overall-equation of girls 634 m and height and weight adjusted 639 m).

In boys < 13 years, age significantly predicted the 6MWD (p = .000, DW 1.539) and neither height nor weight added to age to predict the 6MWD in pre-teenage boys. In adolescent boys ≥ 13 years, age remained the only significant predictor of the 6MWD, weight and height did not add (p = .031, DW 1.789, Table 3).

A 14-year-old boy has a predicted walk distance of 660 m (to compare: overall equation for boys 671 m).

### Impact of vital signs and physical activity scores to predicted 6MWD

Univariate correlation revealed that beside age, height and weight, also exercise HR, MAP at rest and exercise and PAS significantly correlated with the 6MWD. However, gender-separated analysis revealed that PAS correlated with the 6MWD only in boys. Inclusion of vitals at rest and exercise and the PAS in the stepwise regression model in addition to age, weight and height revealed that only exercise HR added information to age and anthropometrics in gender-specific analysis (Table 3).

If this extended model was analysed in gender-specific before puberty, we found that in girls, the exercise HR and height were significant predictors of the 6MWD, whereas in boys, exercise HR, age, and weight were significant predictors (Table 3).

In adolescents, this extended model revealed that in girls the rest and exercise HR, weight, height and PAS all significantly added to the prediction equation, whereas in adolescent males only exercise HR and age were significant predictors of the 6MWD.

## Discussion

**Figure 4:**
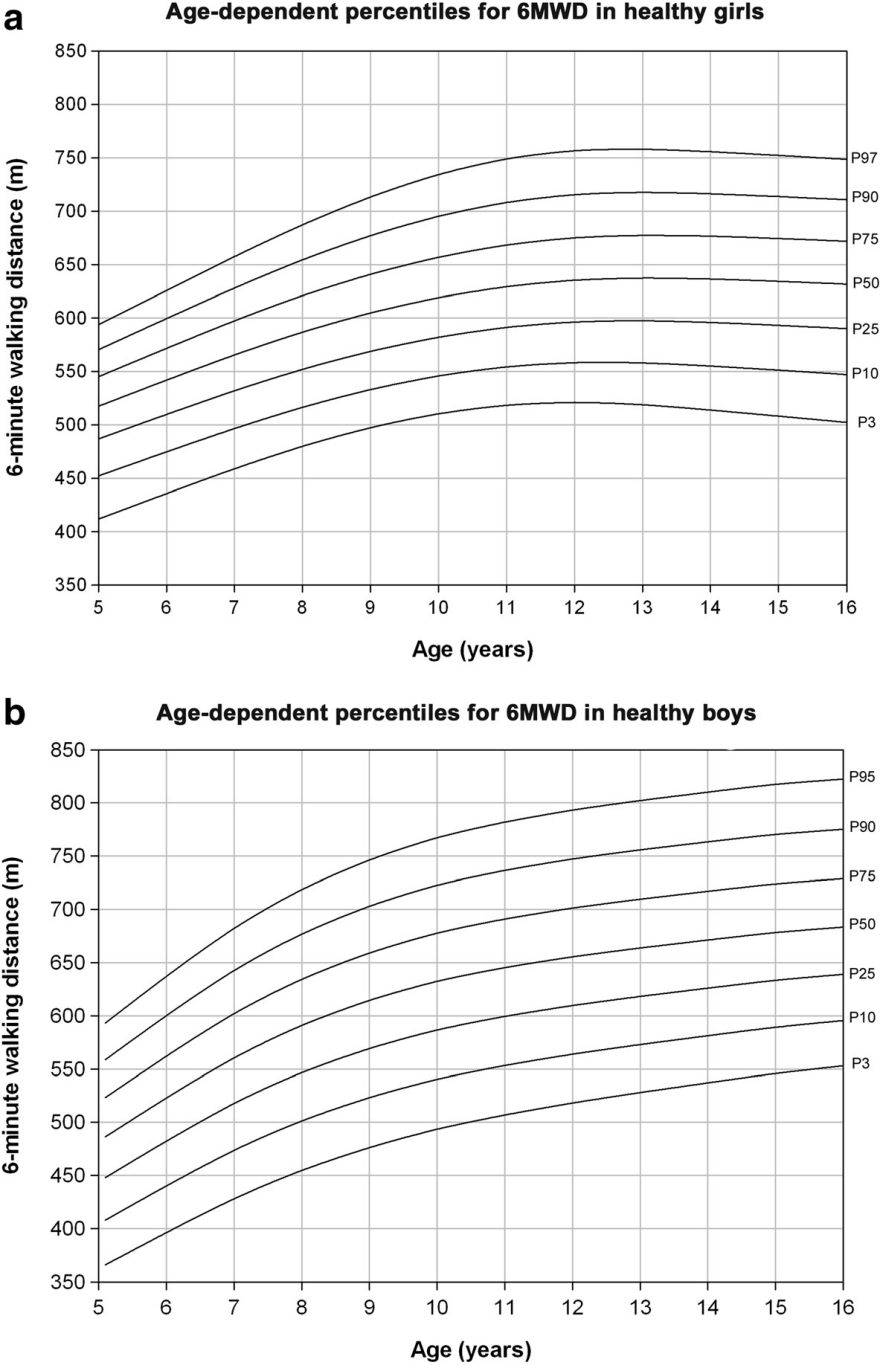
**Shows the development of gender-related 6-minute walking distance in children from 5 to 16 years of age illustrated as percentiles. (a)**. Age-dependent percentiles for 6-minute walking distance (6MWD) in healthy Caucasian girls. **(b)**. Age-dependent percentiles for 6-minute walking distance (6MWD) in healthy Caucasian boys.

Our study shows that the 6MWD in healthy children and adolescents is feasible. The 6MWD was clearly related to gender and age. On the basis of our study we provide gender-specific, age-adjusted reference equations and percentile curves (Figure 4a and 4b) for the 6MWT in healthy children and adolescents living in Switzerland.

Multivariate stepwise models revealed that weight and height may be taken into account; however, their value added is mostly limited and depends on age-subgroup analysed. Beside age and anthropometrics, the HR achieved at peak exercise added to predict the 6MWD, whereas questionnaire assessed physical activity only weakly contributed in post-pubertal girls.

The 6MWT has become a standard in clinical practise and research as a simple tool to assess exercise performance, function and response to treatment in adults with cardiorespiratory disorders [1]. In analogy to the adult population, the 6MWT has become increasingly important in children with cardiorespiratory disorders and it has been shown to be reproducible, reliable and valid [14]. In adults it is still controversial if the absolute 6MWD is best to assess performance and outcome or if expressing the 6MWD in per cent predicted would be better [9]. Reference equations for the 6MWD in adults are available since over a decade [7]. However, they failed to date to show a clear advantage over absolute values. Current guidelines state that the 6MWD is influenced by age, sex, weight, height and motivation, but despite this, absolute thresholds around < 330–380 m are mostly mentioned to be predictors of a poorer prognosis [1,15,16]. Despite this lack of proven clinical validity to express the 6MWD in % predicted in adults, it is obvious that in children and adolescents the influence of age and/or anthropometrics by far outreaches their potential influence on 6MWD compared to adults. Therefore, reference equations for the 6MWD in children and adolescents are of importance for clinicians and researchers treating and assessing children with cardiorespiratory disorders. Li et al. performed 6MWT in healthy children aged 7–16 years in Hong-Kong and found a strong correlation with height [6]. However, Asian children are known to have different anthropometrics compared to Caucasian counterparts. In our Swiss and purely Caucasian collective, we found some influence of height, mainly in prepubertal girls, and weight, mainly in adolescent women. However, age alone was a significant predictor of the distance walked overall and in different subgroups. Lammers et al. performed 6MWT in children aged 4–11 years in the UK and found that in this collective with 83% Caucasians age alone accounted for 41% of the variation in 6MWD, if weight and height were added, 44% could be explained [11]. In our collective, age alone was a significant predictor of the 6MWD overall, gender-specific overall and separately addressed for prepubertal and post pubertal children and adolescents. If height and weight were co-analysed with age in a multivariate stepwise model we found that age alone performed similarly well in gender-specific and overall analysis (Table 3). Reference equations including weight and height might be taken into account for children with anthropometrics at the margins of the normal distribution, e.g. extremely thin, obese, small or large. Therefore we were able to plot age-adjusted normal percentile curves for the 6MWD for children and adolescents and can recommend their use in everyday practice (Figure 4a and 4b). These curves may help to visualize a sick child’s performance in relation to healthy coevals and to observe the performance over time, as it is known for many percentile curves in paediatrics, such as weight and height. In addition, we can provide simple age-adjusted, gender-specific reference equations to assess the % predicted of the 6MWD in girls and boys (Table 3).

In order get insight into the performance of children compared to adolescents we did subgroup analysis by separating girls at an age cut-off of 12 and boys with 13 years. We choose these cut-off points according to assumed midpuberty at around this age. However, further analysis revealed that taking a cut-off point a year earlier or later would not have changed the main results. In females we found that height significantly predicted the 6MWD in prepubertal girls, whereas in adolescents, weight had to be taken into account. We found that anthropometrics did not add to calculate reference values of the 6MWD in boys and adolescent males.

**Figure 5:**
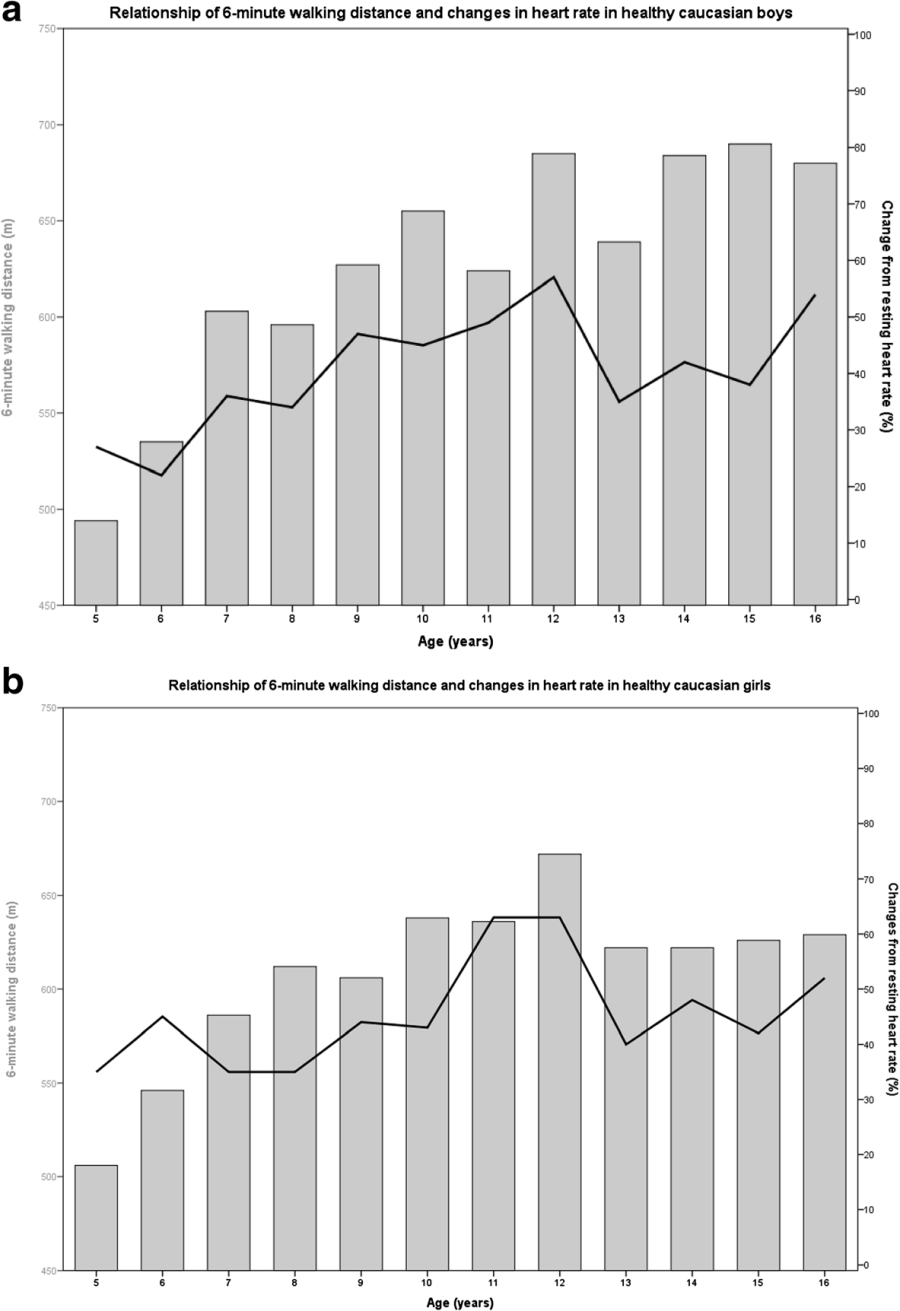
**Shows the 6-minute walking distance walked by children from 5 to 16 years of age and the related physical effort to achieve this distance. (a)**. Relationship of 6-minute walking distance (6MWD) and changes in heart rate in healthy Caucasian boys. **(b)**. Relationship of 6-minute walking distance (6MWD) and changes in heart rate in healthy Caucasian girls.

A further interest of this study was to address the impact of HR, BP, SpO_2_ and reported physical activity assessed by a score on the 6MWD. According to normal children’s development, resting BP slightly increased and HR tended to decrease with age. The BP slightly increased with exercise (mean increase 7 mmHg), the exercise HR increased overall by a mean of 40%. As others [11,17], we found a slightly higher exercise increase in HR in girls compared with boys. Interestingly, we found that the HR achieved at peak 6MWD (exercise HR) significantly added to gender and age to calculate normal 6MWD, even if equations were calculated separately for the subgroups before and after puberty. The degree of HR increase during exercise most probably reflects a higher effort (Figure 5a and 5b). These findings are novel and indicate that the effort taken expressed by exercise HR significantly influences the 6MWD.

In order to get information about physical activity and fitness of the healthy children investigated, we asked them or their legal representatives about participation in sport and other physical activity and scored this with numbers (from 0 to 4, no up to 4 additional weekly activity hours). However, this PAS added significant information to predict the 6MWD only in adolescent girls. Potentially, the questionnaire to assess this PAS did not really reflect physical activity in everyday life. It may well be that some children were very active outdoors in gardens and playground without achieving a high PAS as they did not count outdoor playing to Sports activity. The better performance of this score in adolescents may point towards this direction.

Our work has the following limitations: In our study, the 6MWD was measured only once in every child. So we cannot provide information on the test – retest reliability. We did not include an instrument to measure specific motivation in our study. As we included several children and adolescents of each age group, we believe that the inter-individual differences in motivation would somehow equilibrate around the average. Although we tried to include consecutive children living in different areas without any selection criteria, we cannot exclude a selection bias with potentially more motivated subjects being investigated by us. As we did only the 6MWD test as described in the methods, we do not know the relationship of our test to other submaximal exercise test such as cadence-based estimates or a method including stride length [10,18]. A head-to-head comparison of different tests would be interesting, but also logistically demanding as performing more than one test would potentially be biased by the time sequence. We did also not simultaneously assess other parameters of physiology, such as lung function tests. Peripheral oxygen saturation was normal and remained normal during the test in all healthy children as expected. Our normative values and reference equations are only usable for the age group we investigated. Thus, they will not apply for very young children and toddlers, in which a 6MWD test might as well be difficult to perform. Despite these limitation, in our experience, the present form of the 6MWD is easy performable and utile as comparative measures in the management and follow-up of children and adolescents with cardiorespiratory diseases.

## Conclusions

In summary, in this study we can provide sound reference equations and age-adjusted percentile curve to assess the predicted 6MWD derived from a well-powered and gender-adjusted Swiss cohort. We could show that overall, age was the most important predictor, especially for prepubertal children and boys, anthropometrics significantly add mainly in girls. Exercise HR significantly adds to predict the 6MWD and can be included to calculate effort-adjusted walk distances.

## Abbreviations

6MWD: 6-minute walking distance; 6MWT: 6-minute walk test; BMI: Body mass index; BP: Blood pressure; DW: Durbin-Watson test; HR: Heart Rate; MAP: Mean arterial pressure; PAS: Physical activity score; SD: Standard Deviation; SpO2: Peripheral oxygen saturation.

## Competing interests

The authors declare that they have no competing interests.

## Authors' contributions

All authors had full access to the data and read and approved the final manuscript. *SU* developed the concept of this study, was involved in analysing the data, carried out the statistical analysis and participated in the interpretation, the critical revision of the study and final approval of the manuscript. *FFH* was involved in acquisition of data, analysis and interpretation of data, drafting the manuscript and revising it critically for important intellectual content and provided final approval of the version to be published. *UT* was involved in acquiring the study subjects, taking care for the children during the study period, supervising the data acquisition and performing the tests. *MF* and *SK* made substantial contributions to the current study and publication, participating in the study process, analysis of data, development and revisions of the manuscript and has approved the final manuscript draft. *RS* was involved in the interpretation of data and critical reading and revision of the draft manuscript. *MAF* was involved in analysing the data, carried out the statistical analysis and participated in the interpretation, the critical revision of the study and final approval of the manuscript. All authors read and approved the final manuscript.

## Pre-publication history

The pre-publication history for this paper can be accessed here:

http://www.biomedcentral.com/1471-2466/13/49/prepub
